# Clinical Considerations for Crown Lengthening: A Comprehensive Review

**DOI:** 10.7759/cureus.72934

**Published:** 2024-11-03

**Authors:** Mohammad Qali, Hashem Alsaegh, Samaa Alsaraf

**Affiliations:** 1 Department of Surgical Sciences, College of Dentistry, Health Sciences Center, Kuwait University, Kuwait, KWT; 2 Department of Dentistry, Ministry of Health, Kuwait, KWT

**Keywords:** biologic width, crown lengthening, gingivectomy, oral surgery, osseous

## Abstract

Clinical crown lengthening is a surgical procedure that involves the manipulation of gingival tissue, designed to expose sound tooth structure for restorative purposes by apically repositioning the gingival tissue, with or without the removal of alveolar bone. Crown lengthening may be subdivided as functional or esthetic, according to the purpose of treatment. Functional crown lengthening pertains to the exposure of subgingival caries, fractures, or to avoid invading the biologic width. Whereas esthetic crown lengthening, typically performed in the anterior sextants, aims to lengthen short anterior teeth or refine an uneven gingival contour. Crown lengthening is indicated to address restorative needs, increase clinical crown height, access subgingival caries or perforations, create a ferrule for restorations, or relocate restoration margins. This review manuscript outlines different crown lengthening techniques. Each technique is approached depending on patient-related factors (biologic width, remaining tooth structure, surrounding keratinized tissue, bone, and teeth) and the clinician’s perspective. The importance of reaching a correct diagnosis through meticulous examination of the periodontal condition is key in employing the correct procedure to be conducted. This assures both the practitioner and the patient that the treatment plan will achieve the desired outcome.

## Introduction and background

The concept of crown lengthening was first introduced in 1962 by Cohen DW. This procedure was founded on the principle of preserving sufficient keratinized gingiva around the tooth and establishing a sufficient amount of biologic width [1]. The clinical crown refers to the portion of the tooth that extends beyond the gingiva or mucosa. When the crown is short due to the destruction of the occlusal or incisal surface, or because of a coronally positioned gingival margin, restoring it presents notable challenges. This can impede achieving sufficient resistance and retention, compromise aesthetic goals, and limit access to subgingival caries [2]. Nowadays, dentists routinely face clinical decisions regarding teeth affected by extensive caries or subgingival fractures. Clinicians should evaluate clinical findings alongside patients’ concerns to determine tooth restorability, i.e., whether to extract or restore the affected teeth [3]. Therefore, the restorability of a tooth may be guided by the amount of healthy supracrestal tooth structure that remains and the amount of ferrule that the tooth still possesses [4].

The concept of the dental ferrule was first proposed by Rosen H and Partida-Rivera M in 1961 [5]. The ferrule is defined as a 360-degree metal collar of the crown that encircles the parallel walls of dentin, extending coronally beyond the shoulder of the preparation [6]. It is widely known today that achieving 1.5-2 mm of ferrule and 4.5 mm of supra-alveolar tooth structure is essential for long-term restorative success; this difference includes approximately 2 mm for biological width, and when these criteria are not met, crown lengthening surgery is often performed [6,7].

Clinical crown lengthening is defined as a surgical procedure designed to expose sound tooth structure for restorative purposes by apically repositioning the gingival tissue, with or without the removal of alveolar bone [8]. Crown lengthening surgery is categorized as either functional or esthetic. Functional crown lengthening pertains to the exposure of subgingival caries and fractures. Whereas esthetic crown lengthening, which occurs in the anterior sextants, focuses on the treatment of excessive gingival display caused by delayed passive eruption. This esthetic procedure corrects the appearance of short clinical crowns while preserving the biologic width [3].

To practice crown lengthening with expertise, practitioners must understand the concept of biologic width, as well as the indications, contraindications, the difference between esthetic and functional crown lengthening, different crown lengthening techniques, and other essential principles involved. Therefore, this manuscript will serve as a comprehensive guide and a reference for each practitioner willing to execute crown lengthening procedures.

## Review

Biologic width (supracrestal attachment)

**Figure 1:**
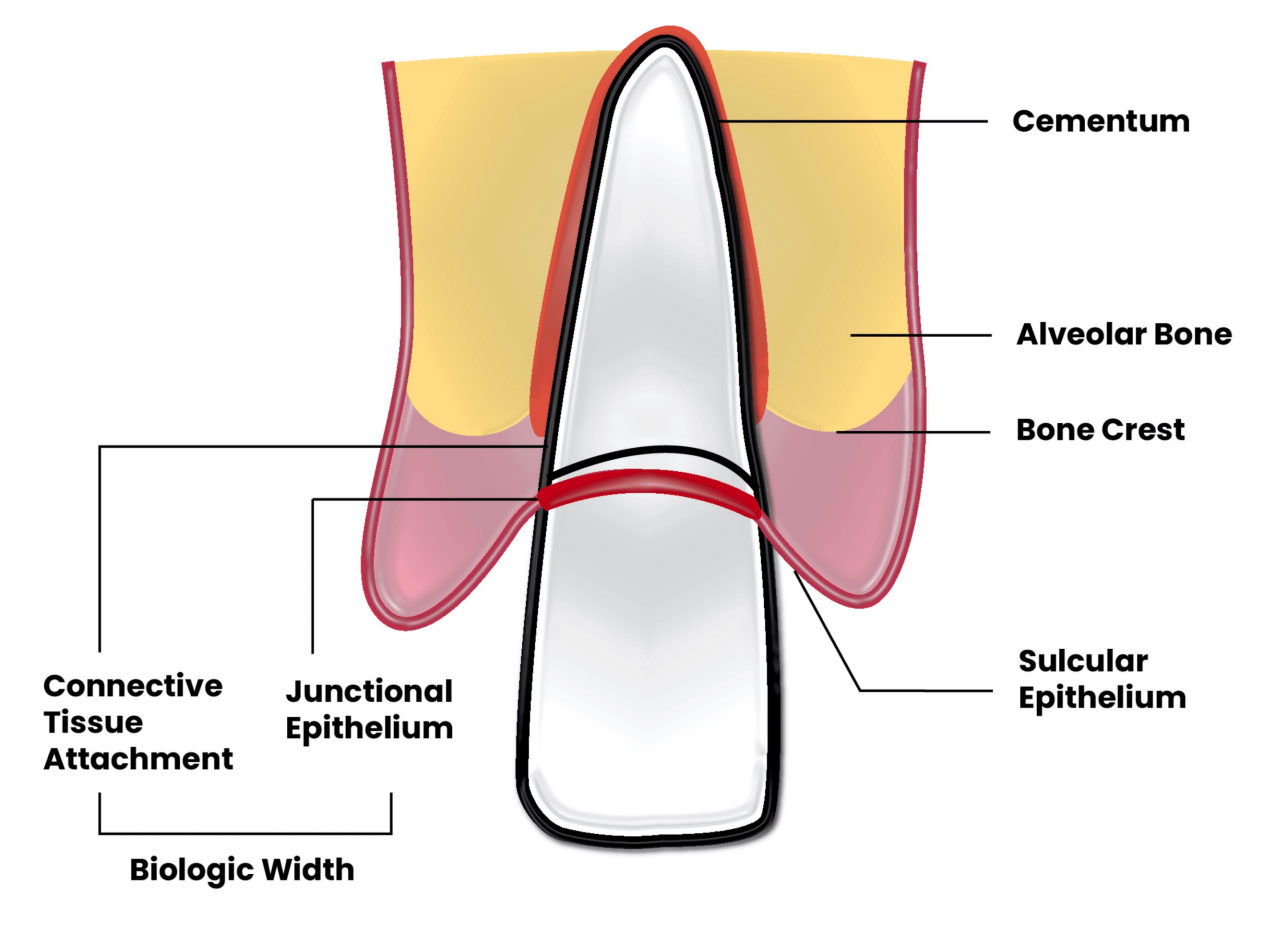
A schematic illustration depicting the components of the periodontium and the biological width space. Figure illustrated by Abdullah Khalil.

Gargiulo defined biologic width as the combined dimension of the epithelial and connective tissue attachment to the root, which he determined to average 2.04 mm (a length of epithelial attachment of 0.97 mm, and a length of supracrestal connective tissue of 1.07 mm). In other words, it is the dimension of the soft tissue attached to the portion of the tooth coronal to the alveolar bone crest [9,10] (Figure 1). Also, Vacek JG et al., in 1994, suggested that biologic width increases anteroposteriorly from 1.75 mm to 2.08 mm [11].

When the biologic width is violated, the body attempts to reestablish it through osseous resorption. This pathological process is believed to often result in chronic inflammation, bone loss, and periodontitis [12]. Recommendations for the extent of supracrestal tooth exposure during crown lengthening surgery vary. Achieving 1.5-2 mm of ferrule and 3 mm of supra-alveolar tooth structure is essential for long-term restorative success; other studies suggest this facilitates biologic width formation and ensures prosthesis retention (Figure 1) [13,14].

Indications of crown lengthening

The indications for crown lengthening include addressing restorative needs, such as obtaining access to subgingival caries, acquiring a ferrule for restorations, and relocating restoration margins that impinge on the biological width. Other indications include increasing clinical crown height lost due to caries, fractures, or wear and accessing a perforation in the coronal third of the root. Aesthetic concerns such as short teeth, uneven gingival contours, and a gummy smile may also be resolved by crown lengthening [15].

Contraindications of crown lengthening

Contraindications for crown lengthening include an inadequate crown-to-root ratio (normal 1:2), non-restorability due to caries or root fractures, esthetic compromise, high furcation involvement, and low predictability of outcomes. Crown lengthening is also contraindicated in cases with an inadequate tooth-to-arch relationship, potential compromise to the adjacent periodontium or aesthetics, insufficient restorative space, and lack of maintainability and root proximity [3,16].

Pre-surgical considerations of crown lengthening

A key consideration when planning for crown lengthening surgery is evaluating the width of the keratinized gingiva, the distance from the cementoenamel junction (CEJ) or restoration margin to the crestal bone, periodontal pockets, the crown-to-root ratio, root anatomy and relationships, gingival biotype, thickness of the buccal alveolar bone, and supracrestal gingival tissue [8].

Esthetic crown lengthening 

Smile aesthetics play a major role in the perception of a person’s attractiveness and confidence, as well as other social parameters. Simply restoring lost tooth structure is considered faulty, and patients now expect anterior rehabilitations to also be aesthetically pleasing [17]. Some of the issues that patients might complain about include excessive gingival display or a gummy smile, which might have a negative impact on the esthetic appearance of the patient [18,19]. This is caused by the hyperactivity of the elevator muscle of the upper lip, vertical downward growth of the maxilla, gingival enlargement, and/or altered passive eruption. Managing excessive gingival display may require one or multiple treatment approaches depending on its underlying cause.

When the underlying cause of excessive gingival display results from altered passive eruption or gingival enlargement, periodontal surgeries can provide an effective correction [20]. Crown lengthening procedures, such as the apically positioned flap and gingivectomy with bone re-contouring when needed, can enhance excessive gingival display and uneven gingival contours caused by altered passive eruption [3,15,21].

Several studies have examined classification systems for altered passive eruption. Coslet JG et al. identified two primary types: Type I, characterized by excessive gingival tissue with the gingival margin positioned incisal or occlusal to the CEJ, and type II, which involves normal amounts of gingival tissue with the gingival margin within the typical range from the CEJ, where all attached gingival tissue is situated on the anatomic crown, and the mucogingival junction is at the level of the CEJ [18,22,23].

**Figure 2:**
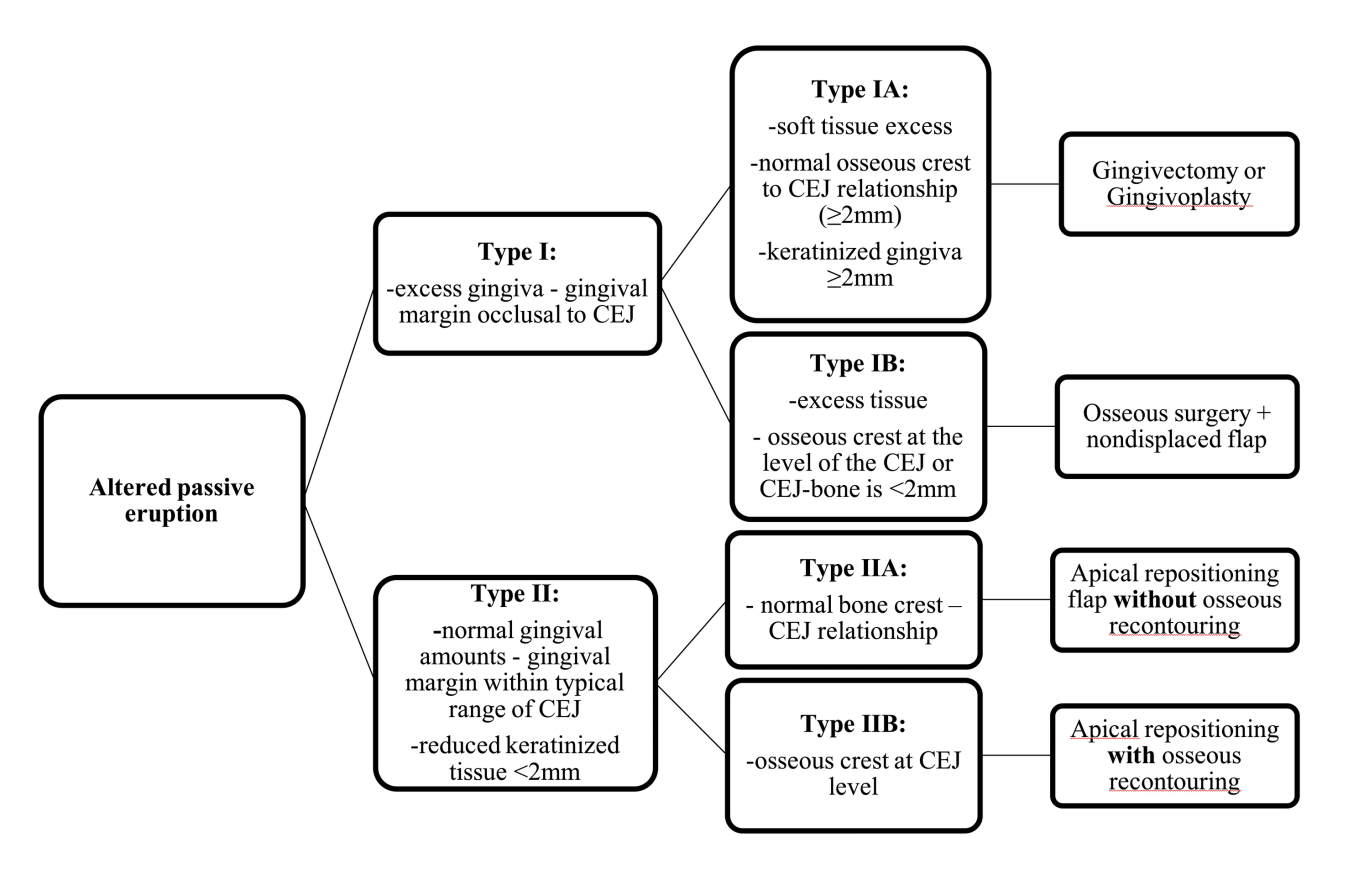
Subtypes of altered passive eruption: descriptions and management strategies.

Depending on the clinical and radiographic findings, four approaches can be employed for this procedure (Figure 2). Type I altered passive eruption is subdivided into two categories: Type IA is characterized by soft tissue excess with a normal osseous crest to CEJ relationship (adequate keratinized gingiva ≥2 mm, and CEJ-bone is ≥2 mm), requiring only a gingivectomy or gingivoplasty for correction. Type IB involves both excess tissue and the osseous crest at the level of the CEJ or CEJ-bone is <2 mm, necessitating osseous recontouring. Type IIA includes patients with reduced keratinized tissue (<2 mm) and a normal bone crest to CEJ relationship, requiring apical repositioning flap without osseous recontouring. Type IIB features inadequate keratinized tissue and an osseous crest at the CEJ level, requiring both apical repositioning of the flap and osseous recontouring to achieve aesthetic results without biologic width impingement [18,22,24].

According to principles of wound healing and related studies, tissue maturation and healing take 4 to 6 weeks following a gingivectomy procedure. If a buccal flap was elevated and bone exposed, tissue maturation and stabilization require 8 to 12 weeks. In cases where bone was removed, at least a 6-month period is necessary for soft-tissue stabilization. Additionally, a secondary surgery may be required 6 to 12 weeks later to refine the aesthetic outcome of the smile line (Figure 2) [4,25-29].

Functional crown lengthening 

Functional crown lengthening is performed to avoid impingement on the biologic width, facilitate the creation of a ferrule effect, reveal subgingival caries and root fractures, and enhance the amount of supragingival tooth structure to improve the retention of future restorations [30].

**Figure 3:**
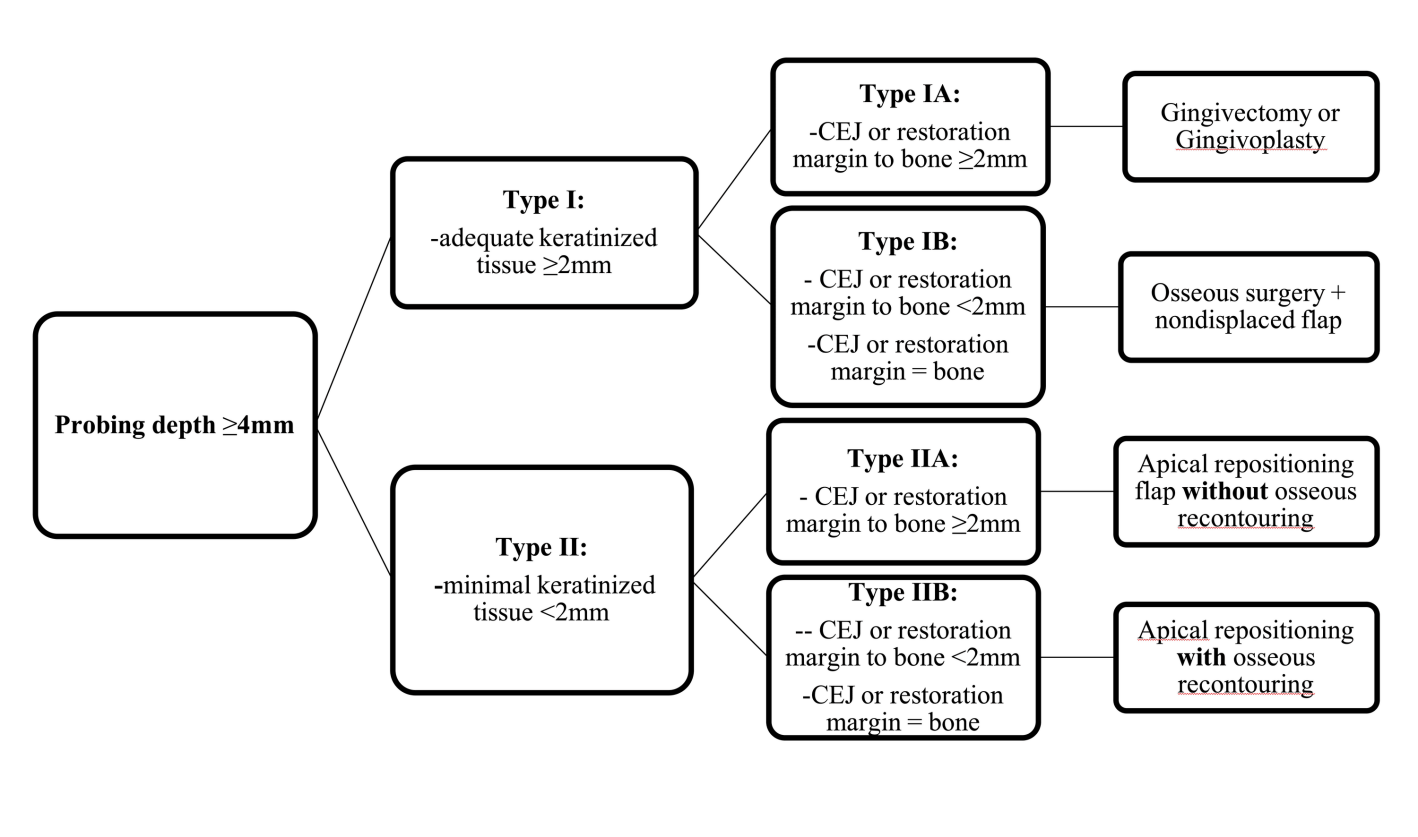
How to approach functional crown lengthening: types and management of each.

Depending on the clinical and radiographic findings, five approaches can be employed for this procedure. For a tooth with a probing depth of ≥4 mm and sufficient keratinized gingiva, a gingivectomy or gingivoplasty is performed (first approach). If keratinized gingiva is insufficient, an apically positioned flap is used (second approach). When bone removal is necessary to establish biologic width, an apically positioned flap with osseous surgery is indicated (third approach). In cases where preserving adjacent structures and interdental papillae is crucial, such as with a fractured tooth in the anterior region, orthodontic forced eruption is undertaken, potentially followed by crown lengthening (fourth approach). If crown lengthening would result in an unfavorable crown-to-root ratio or cause more harm to the surrounding periodontium of neighboring teeth, extraction is recommended, with a dental implant replacement considered later (fifth approach) [12,31-35]. Figure 3 shows clinical assessment and planning prior to initiating a functional crown lengthening procedure.

Techniques of clinical crown lengthening

Nowadays, several techniques are being implemented to achieve clinical crown lengthening. These include gingivectomies, apically positioned flaps with or without osseous reduction, and forced eruption alone or in conjunction with surgical crown lengthening [36-39].

Gingivectomy 

Gingivectomy is a surgical procedure that involves removing the supracrestal gingival tissue while preserving the biological width of the supracrestal tissue attachment. This can be done with or without osseous surgery [40]. The clinician excises the gingival tissue, i.e., gingivectomy, by means of a scalpel, electrosurgery, chemosurgery, and laser [14].

**Figure 4:**
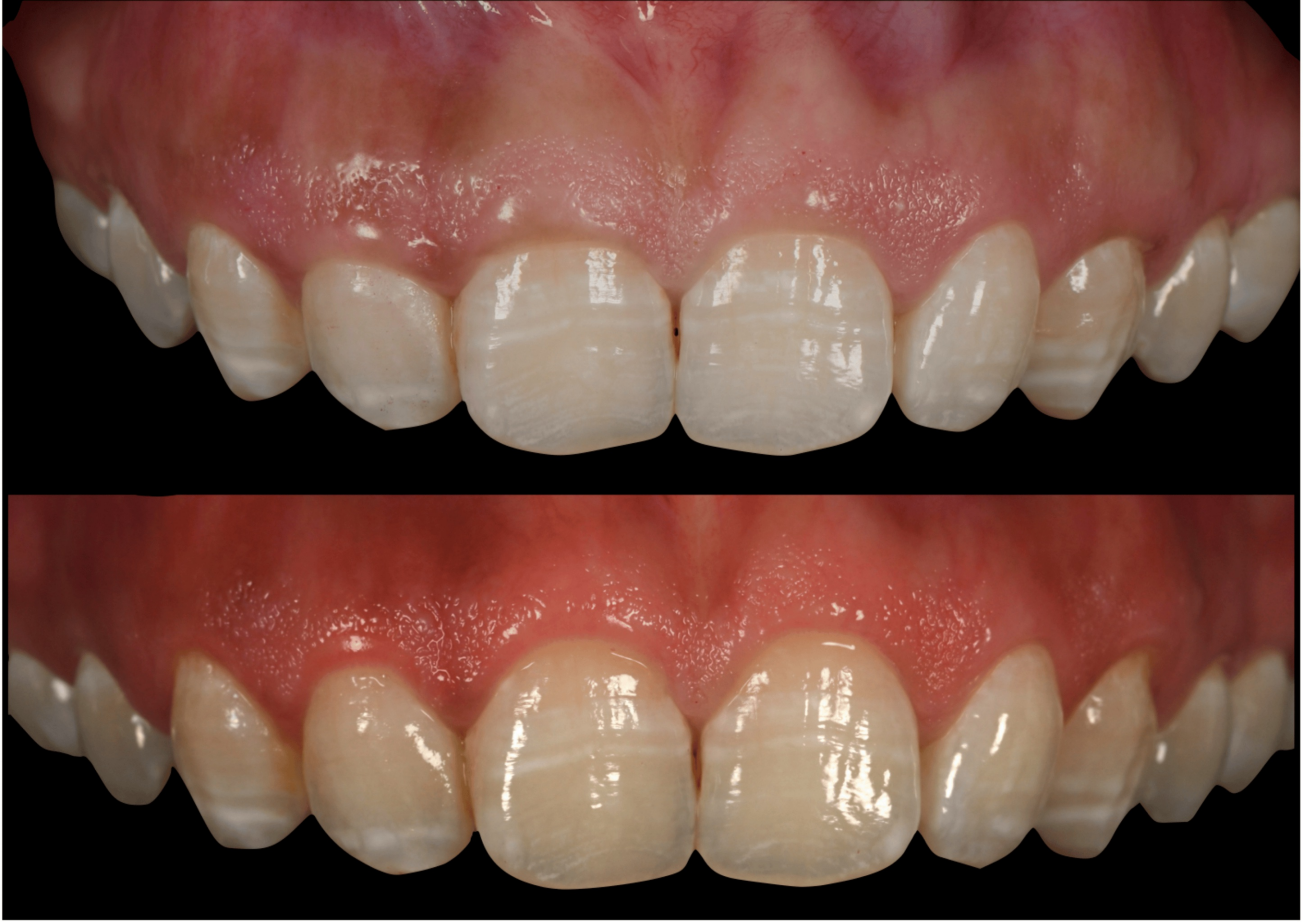
Crown lengthening by gingivectomy without osseous reduction. Dr. Qali's clinical case (first author); permission granted.

**Figure 5:**
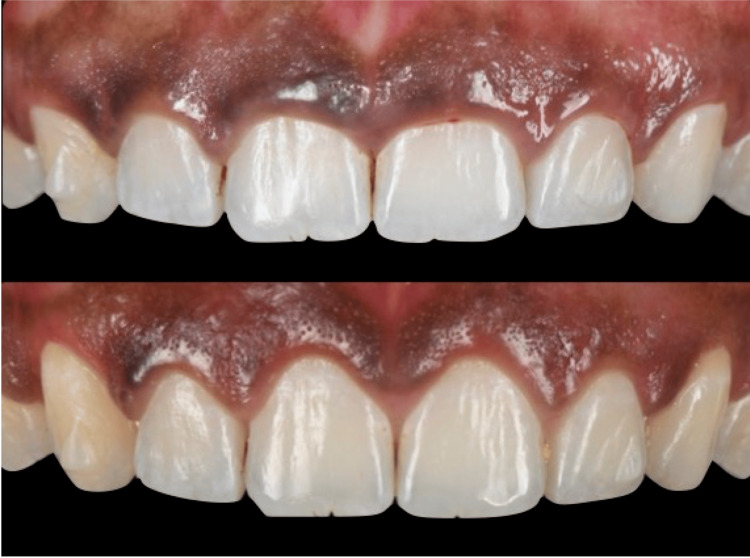
Crown lengthening by gingivectomy with osseous reduction. Dr. Qali's clinical case (first author); permission granted.

Regardless of the tool used, a gingivectomy could lead to the complete removal of attached gingival tissue. Clinicians must consider the gingival width in an occlusal-apical dimension. Gingivectomy eliminates the excess keratinized tissue. The need for osseous reduction along with the gingivectomy is determined by the underlying bone crest. If the bone crest is less than 3 mm away from the level of the gingival margin, gingivectomy with osseous reduction is indicated. However, if the underlying bone crest is 3 mm or more from the new gingival margin, osseous reduction is not necessary (i.e., gingivectomy without osseous reduction). Maynard and Wilson recommended maintaining at least 3 mm of attached gingiva for subgingival restorative therapy. If a gingivectomy would result in a postoperative gingival width of less than 3 mm, an apically positioned flap without osseous surgery should be considered as an alternative [21,31,32,41]. If the initial amount of gingiva is limited, the dentist may opt for a sulcular incision positioned at the osseous crest. This approach not only conserves gingival tissue but also enhances the width of attached gingiva following the healing process (Figures 4-5) [42].

Apically Positioned Flap With or Without Osseous Resection

**Figure 6:**
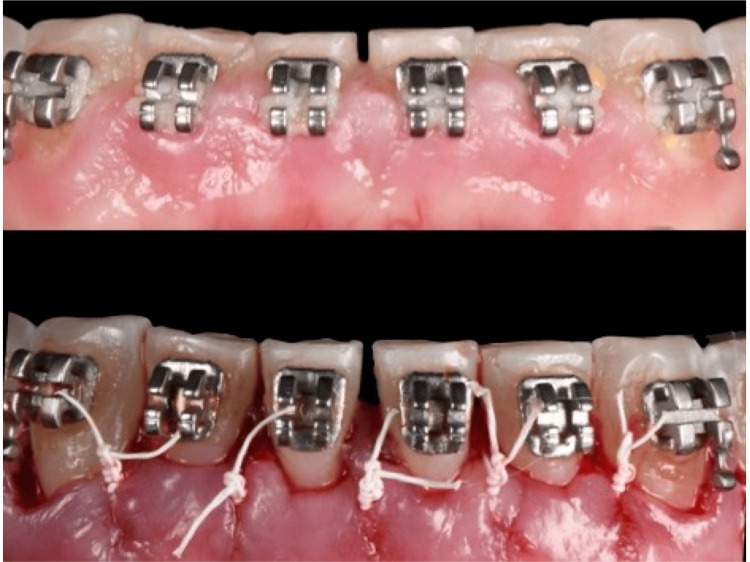
Crown lengthening by apically positioned flap without osseous reduction. Dr. Qali's clinical case (first author); permission granted.

**Figure 7:**
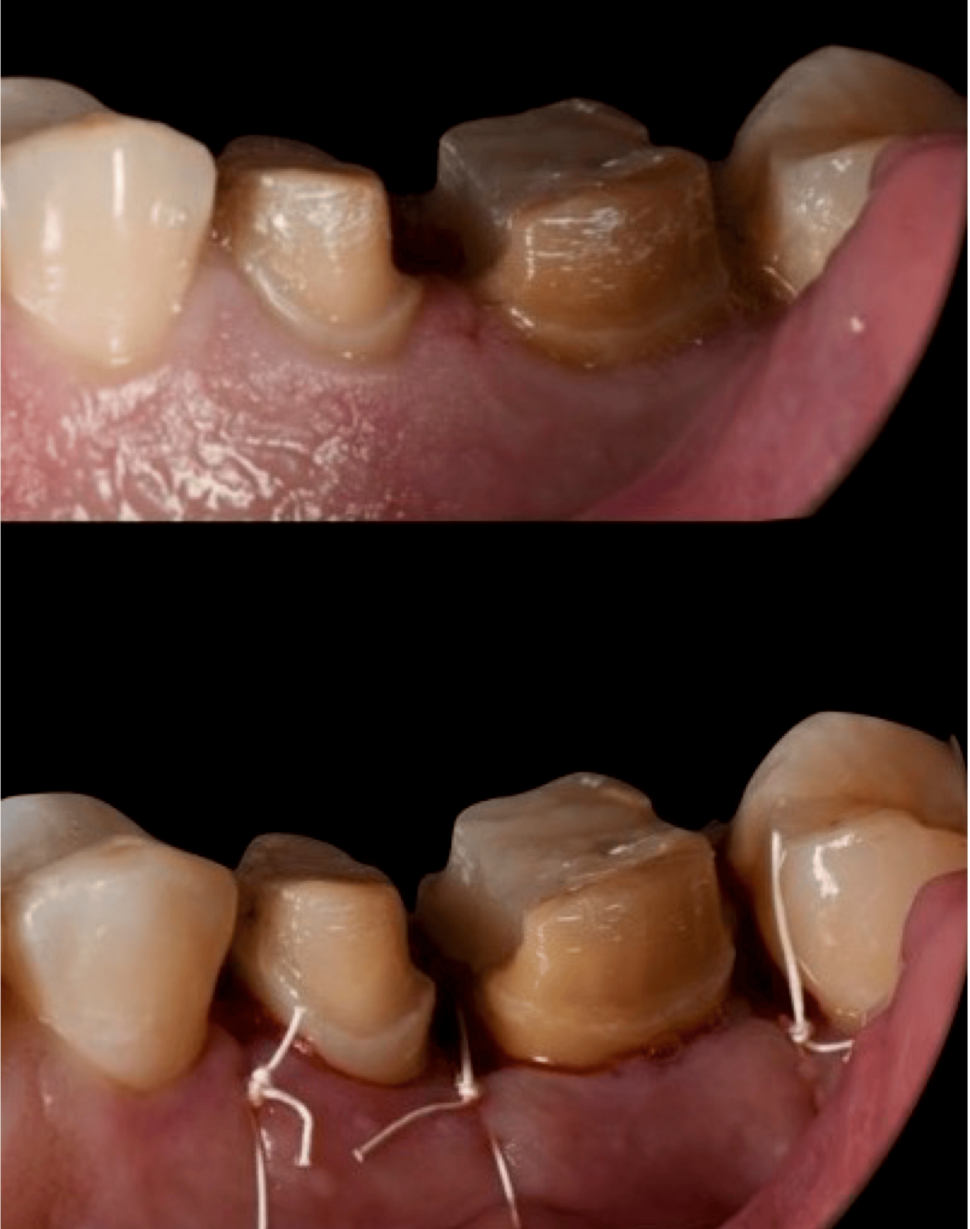
Crown lengthening by apically positioned flap with osseous reduction. Dr. Qali's clinical case (first author); permission granted.

When visualization of the underlying bone crest is necessary, it is advisable to use an apically positioned flap with bone resection, particularly when the osseous level is less than 3 mm from the gingival resection level. In such cases, a gingivectomy may lead to postsurgical biologic width dimensions and probing depths reverting to their pre-surgical values, resulting in greater tissue rebound and reduced crown exposure compared to using an apically positioned flap combined with ostectomy (Figures 6-7) [43].

Garber DA et al. stated, “The tissue is the issue, but the bone sets the tone” [44]. Osseous resection encompasses ostectomy and osteoplasty. Unlike osteoplasty, ostectomy involves the removal of supporting bone connected to the root surface via the periodontal ligament. Various instruments can be utilized for osseous resection, including handpiece burs, hand chisels, piezoelectric tips, and potentially Er:YAG laser [45,46]. The bone structure must be contoured to establish a scalloped positive architecture, facilitating the formation of a scalloped gingival architecture with minimal pocket depths. In cases where the osseous architecture resulting from osseous resection is negative (reversed architecture), there is a risk of excess gingival tissue rebound during the healing phase following the crown lengthening procedure. The reduction of osseous ledges or exostoses through osteoplasty was first recommended by Schluger in 1949 and later by Friedman in 1955 [33,47]. Flaps are then finally sutured coronally to the alveolar crest at a distance similar to baseline pre-surgical supracrestal gingival tissues.

Following the completion of the surgical procedure, the healing phase commences. Studies have proven that performing an apically positioned flap combined with osseous resection leads to the reestablishment of the biological width at a more apical level [48]. Other studies noted that positioning the margin of the flap at the osseous crest results in an average postoperative vertical gain or rebound of 3 mm in the supracrestal soft tissues [21,49]. However, if the flap margin is positioned at a level more coronal to the newly established osseous crest, rebound in supracrestal soft tissues has been observed (Figures 6-7) [29].

Forced Eruption

Orthodontic extrusion: Orthodontic extrusion is a valuable treatment option in the management of compromised or non-restorable teeth. Periodontal surgical approaches may not be indicated because of the amount of support that needs to be removed from the involved tooth and the healthy periodontium of the adjacent teeth. Additionally, internal and external root resorptions, horizontal fractures, and isolated periodontal defects indicate a forced eruption technique rather than crown lengthening by osseous surgery. Selection of the forced eruption technique depends on root length and shape. For instance, teeth with short, delicate roots are not good candidates because of unfavorable crown-to-root ratios that may be created [50-53]. Orthodontic extrusion can be executed using two approaches. 

The first approach requires the application of low orthodontic force, which gradually extrudes the tooth, causing the alveolar bone and gingival tissue to move along with the tooth. The tooth is extruded until the bone level is slightly above the desired position, accounting for the bone that will need to be surgically removed to correct any attachment issues. Once the tooth reaches this new position, it is stabilized, and a subsequent surgery is performed to adjust the bone and gingival tissue levels [32,38].

**Figure 8:**
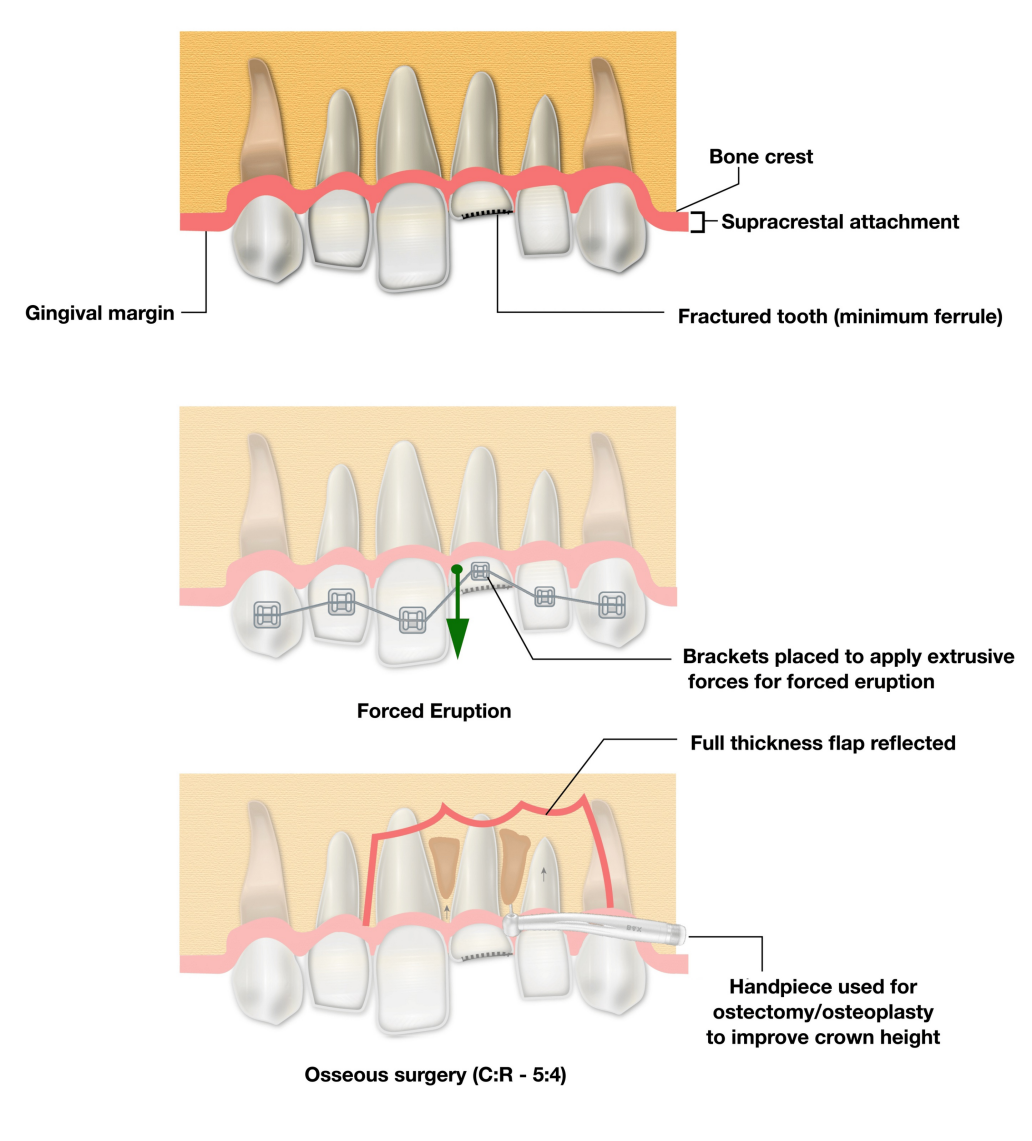
A comparison of crown-to-root ratios reveals that crown lengthening alone results in a ratio of 5:4, whereas extrusion combined with osseous surgery or fiberotomy achieves a ratio of 4:4 when addressing a fractured tooth lacking sufficient ferrule for a full-coverage restoration. Figure illustrated by Abdullah Khalil.

The second approach uses rapid orthodontic extrusion, where the tooth is extruded at a faster rate. During this process, a supracrestal fibrotomy is performed weekly to prevent the bone and tissue from moving with the tooth. In some cases, particularly with rapid extrusion, osseous reduction is unnecessary, and soft tissue can be removed through simple excision. The tooth is then stabilized for at least 12 weeks to ensure the tissue and bone remain in their desired starting position, and any coronal tissue movement can be surgically corrected if needed [12,32,38] (Figure 8).

Surgical extrusion: One of the surgical extrusion techniques is by using periotomes or elevators [54]. This technique can effectively address severely damaged supracrestal tooth structures due to fractures or dental caries. This procedure is also resourceful for salvaging teeth with deep cervical root fractures and caries that are challenging to manage conservatively [39]. This method is particularly useful for cases influenced by iatrogenic factors, especially in the anterior region where aesthetics are crucial. Additionally, it meets aesthetic requirements for maintaining the gingival margin following a crown lengthening procedure in a single anterior tooth and has demonstrated predictable short and long-term outcomes [55,56]. However, during luxation, the clinician must exercise care to mitigate the risk of subsequent root resorption [57,58].

Timing of crown lengthening with orthodontic treatment

The timing of crown lengthening may be before, during, or after orthodontic treatment. The most important limiting factor is the crown height at the beginning. In other words, crown lengthening pre-orthodontic treatment is necessary when the crown height is insufficient for the ideal bracket position [59-60]. Patients may need crown lengthening surgery during orthodontic treatment when the excessive gingival tissue is harboring bacteria and hindering proper oral hygiene. The second step in the decision-making process for the timing of the surgery is the restorative treatment plan. When the patient needs anterior restorative work at the end of the orthodontic treatment or the orthodontist has any doubts regarding the correct incisal edge positions, crown lengthening is advised during the treatment. Prior to restorations, teeth must be positioned correctly mesio-distally and apico-coronally. The apical/coronal position is determined by the level of the CEJ, which sometimes cannot be leveled by the orthodontist due to excessive gingiva, necessitating surgery during the orthodontic treatment (towards the end). If the patient does not need anterior restorative work, the final tooth position is dictated by the orthodontist, and so the crown lengthening procedure is delayed until after debonding. It is often advised to perform crown lengthening procedures after the termination of orthodontic treatment, whenever possible, to avoid a touch-up surgery at the end [61]. Nowadays, clear aligners are becoming more popular. In some cases, crown lengthening is performed prior to bonding for orthodontic treatment to increase the apico-coronal dimensions of the clinical crown to have more predictable tooth movement. Furthermore, this is due to the action of the aligners depending on the size of the tooth itself. Also, gingivectomy may be useful during the treatment if gingival overgrowth is impeding proper aligner seating, which is most important to assure effective treatment.

Maxillary anterior teeth: gingival zenith positions and levels 

The positioning of the gingival margins of the six maxillary anterior teeth is vital for the aesthetic appearance of the crowns. In other words, creating or re-establishing the dental zenith in the maxillary anterior teeth enhances the overall aesthetic outcome [62-64]. For instance, the gingival margin of the two central incisors should align at the same level. Furthermore, the gingival margin of the central incisors should be positioned more apically than that of the lateral incisors, and the gingival margins of the central incisors should match the level of the canines. In addition to that, the contour of the labial gingival margins should replicate the cementoenamel junctions of the teeth [65]. The clinician should always contemplate these details in their treatment to achieve superlative aesthetic outcomes.

Guided crown lengthening 

Surgical guides are typically created as a reference for crown lengthening procedures, particularly in cases involving anterior teeth [66]. Some clinicians opt for the utilization of surgical guides in crown-lengthening procedures, especially when multiple anterior teeth are involved and significant alterations of the gingiva and bone levels are anticipated. The employment of a surgical guide can help prevent excessive or insufficient contouring of the bone and gingiva. Various researchers have documented the use of surgical guides in these procedures to delineate the final gingival margin level and establish the biological width [67-71]. One of the advantages of the template is that it guarantees the suturing of the flap in the proper position, so that its edge coincides with that of the template [72,73].

Achieving aesthetic crown lengthening often requires a collaborative effort, integrating advanced surgical techniques with carefully coordinated orthodontic interventions and, at times, full rehabilitation restorative procedures. Consequently, meticulous case planning, with effective communication not only with the patient but also among clinicians and involved laboratories, is crucial [69].

In the digital dentistry era, mastering appropriate digital hardware and software for case planning can offer various advantages. Each clinician can contribute to the treatment plan, creating a blueprint for predictable and consistent patient outcomes. A virtual plan for the final tooth position in conjunction with the necessary prosthodontic plan minimizes guesswork and repetition. Utilizing digital smile design and digital wax-up facilitates the fabrication of precise 3D printed surgical stents. These steps serve as valuable adjuncts to traditional treatment methods for similar cases. This advancement may be employed in aesthetic crown lengthening. An advanced surgical guide can streamline surgical steps by incorporating not only the soft tissue component but also the 'built-in' supracrestal attachment apparatus (biological width). This guide provides the surgeon/periodontist with a clear preview of the amount of osseous structure resection, enhances communication between the surgeon and the orthodontist/prosthodontist/restorative dentist, and ensures accurate information transfer [74]. Clinicians should aim to be competent in using both the freehand and guided techniques.

## Conclusions

This review manuscript outlines different crown lengthening techniques. Each technique is approached depending on patient-related factors (biologic width, remaining tooth structure, surrounding bone and teeth) and the clinician’s perspective. The purpose of the crown lengthening procedure can be either aesthetic or functional. Aesthetic crown lengthening is executed in the maxillary anterior region to improve smile aesthetics. On the other hand, functional crown lengthening is performed to enhance restorative work by improving the remaining tooth structure, i.e., the ferrule effect. The importance of reaching a correct diagnosis through meticulous examination of the periodontal condition is key in employing the correct procedure. This assures both the practitioner and the patient that the treatment plan will achieve the desired outcome.
